# Impact of acute heat stress on mitochondrial function, ultrastructure and cardiolipin distribution in *Arabidopsis*

**DOI:** 10.1007/s42994-024-00151-x

**Published:** 2024-03-15

**Authors:** Yukang Wang, Ronghui Pan, Jianping Hu

**Affiliations:** 1 College of Agriculture and Biotechnology, Zhejiang University, Hangzhou, 310058 China; 2 Michigan State University-Department of Energy Plant Research Laboratory and Plant Biology Department, Michigan State University, East Lansing, MI 48824 USA

**Keywords:** High temperature, Plant heat stress response, Mitochondrial membrane potential, Cristae structure, Cardiolipin redistribution and externalization

## Abstract

Besides providing energy to sustain life, mitochondria also play crucial roles in stress response and programmed cell death. The mitochondrial hallmark lipid, cardiolipin (CL), is essential to the maintenance of mitochondrial structure and function. However, how mitochondria and CL are involved in stress response is not as well defined in plants as in animal and yeast cells. We previously revealed a role for CL in mitochondrial fission and in heat stress response in *Arabidopsis*. To further determine the involvement of mitochondria and CL in plant heat response, here we treated *Arabidopsis* seedlings with varied lengths of acute heat stress. These treatments resulted in decreases in mitochondrial membrane potential, disruption of mitochondrial ultrastructure, accumulation of mitochondrial reactive-oxygen species (ROS), and redistribution of CL to the outer mitochondrial membrane and to a novel type of vesicle. The level of the observed changes correlated with the severeness of the heat stress, indicating the strong relevance of these processes to stress response. Our findings provide the basis for studying mechanisms underpinning the role of mitochondria and CL in plant stress response.

Dear Editor,

Mitochondria are highly involved in stress response (Xue and Hua 2017; Bar-Ziv et al. 2020; Eckl et al. 2021). In mammalian cells, mitochondrial stress induces mitophagy and programmed cell death (PCD) (Kubli and Gustafsson 2012). In plant cells, mitochondria are also important in stress responses (Huang et al. 2016; Barreto et al. 2022). However, how mitochondria are involved in plant stress responses, through processes such as mitophagy and PCD, remains largely ambiguous.

The phospholipid cardiolipin (CL) is a signature lipid of mitochondria with fundamental importance in mitochondrial energy metabolism, ultrastructure maintenance, and fission/fusion (Acoba et al. 2020; Pan et al. 2014a; Pan and Hu 2015). Based on the studies in animal cells, the highly unsaturated CL, which is normally enriched in the mitochondrial inner membrane, is susceptible to mitochondrial oxidative stress (Wiswedel et al. 2010). In mammals, mitochondrial stress causes cytochrome c-mediated CL peroxidation, followed by the externalization of CL to the surface of the mitochondrial outer membrane, where CL triggers the initiation of mitophagy, or even PCD (Pizzuto and Pelegrin 2020; Acoba et al. 2020; Dudek 2017). During PCD, mammalian cells were shown to deplete CL from the mitochondria, which can be blocked by antioxidants (Kirkland et al. 2002), and relocate CL to other subcellular membranes (Sorice et al. 2004).

In plants, the acyl chains of CL are highly enriched in unsaturated lipid species, like linoleic and linolenic acids, suggesting its sensitivity to oxidative stress (Zhou et al. 2016). Disruption of cardiolipin synthase in *Arabidopsis* led to severely impaired plant growth and development, as well as defects in mitochondrial functions, including respiration and the tricarboxylic acid cycle, and in mitochondrial morphogenesis, including the balance of organelle fission and fusion and cristae structure (Katayama and Wada 2012; Pineau et al. 2013; Pan et al. 2014a; Petereit et al. 2017). CL was shown to be important for plant tolerance to stresses, including acute heat shock (Pineau et al. 2013; Pan et al. 2014a). However, the importance and behavior of CL during plant stress response is still poorly understood.

As a follow-up study of our previous work, which revealed a role for CL in mitochondrial fission and heat stress response, the current study aims to further determine how acute heat stress impacts mitochondria and CL in plants. First, we incubated *Arabidopsis* seedlings at 65 °C, the same temperature as we used previously for heat stress (Pan et al. 2014a), for 5, 15 and 30 min. The 5-min heat shock did not cause obvious morphological deformity of the plants (Fig. 1A). Plants treated with 15-min heat shock did not show immediate visible damages, but withered after 24–36 h of recovery at room temperature (Fig. 1B). The 30-min heat treatment immediately caused plant death. To determine changes in mitochondrial function after the heat treatment, we assessed the mitochondrial inner membrane potential (MIMP), which is a key indicator of mitochondrial respiratory function, by staining plant cells with the tetramethylrhodamine, methyl ester (TMRM) stain immediately after the heat treatment. Untreated plants showed bright TMRM fluorescence signals, whereas the signals greatly decreased after 5- or 15-min heat stress treatment, and were completely lost after the 30-min heat stress (Fig. 1C). These observations demonstrated that acute heat shock effectively impairs the mitochondrial respiratory ETC function.

**Fig. 1:**
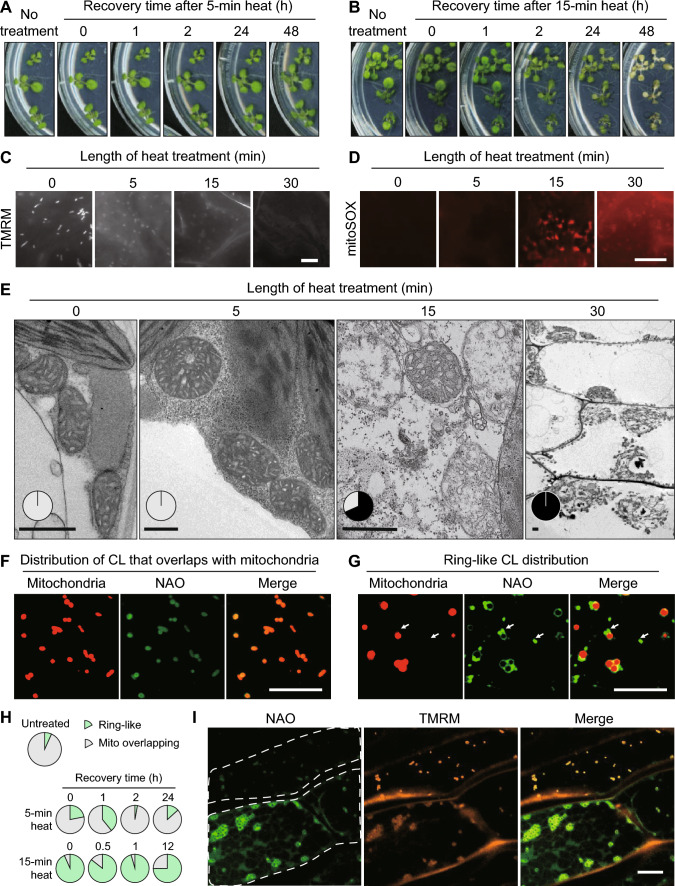
Impact of acute heat stress on mitochondrial function, ultrastructure and cardiolipin distribution. (**A**, **B**) Plant recovery after acute heat shock. Two-week-old *Arabidopsis* plants treated with 65 °C heat for 5 min (**A**) or 15 min (**B**) were left to recover at 22 °C for varied lengths of time. **C** Loss of mitochondrial inner membrane potential after acute heat shock. Epifluorescence images from *Arabidopsis* leaf epidermal cells show mitochondrial inner membrane potential by TMRM (tetramethylrhodamine, methyl ester) staining. Plants were treated with different lengths of heat shock (65 °C). Scale bar, 5 µm. **D** Generation of mitochondrial ROS after acute heat shock. Epifluorescence images of plant leaf epidermal cells stained by MitoSOX™ Red Mitochondrial Superoxide Indicator, which indicates mitochondrial superoxide levels, are shown. Plants were treated with different lengths of heat shock (65 °C). Scale bar, 10 µm. **E** TEM analysis of mitochondrial ultrastructure from *Arabidopsis* leaf tissue after acute heat shock. Plants were treated with different lengths of heat shock (65 °C). Scale bar, 0.5 µm. In the inserted pie charts, the black portion indicates the percentage of mitochondria showing disrupted ultrastructure; the white portion indicates that of mitochondria showing normal ultrastructure. **F**, **G** Confocal images of leaf epidermal cells of 2-week-old *Arabidopsis* plants expressing COX4-YFP stained with the CL-specific dye NAO. Scale bar, 10 µm. CL showed a mitochondrial overlapping distribution pattern in (**F**) and ring-like distribution in (**G**). Arrows indicate possible mitochondrial derived vesicle-like structures enriched in NAO staining. Scale bar, 10 µm. **H** Quantification of CL distribution patterns after heat treatment and different length of recovery time. The white portion indicates the mitochondrial overlapping distribution pattern; the green portion indicates the ring-like distribution pattern. **I** Mitochondria with externalized CL have lower MIMP. Leaf epidermal cells of 2-week-old *A. thaliana* plants expressing COX4-YFP were double stained with NAO and TMRM. Scale bar, 10 µm

Under stress, an increased generation of ROS in the mitochondrial electron transport chain (ETC) results in accumulation of ROS that can cause oxidative damage to mitochondrial proteins, nucleic acids and lipids (Blokhina and Fagerstedt 2010). To determine whether this heat-induced mitochondrial functional disturbance was accompanied with ROS accumulation, we stained the plants with the MitoSOX Red Mitochondrial Superoxide Indicator, which is a cell-permeable fluorogenic probe that selectively targets mitochondria and can be oxidized by local superoxide (Nie et al. 2015).

A minimal level of mitoSOX fluorescence was detected in untreated plants and plants treated with 5-min heat shock (Fig. 1D), which is consistent with the fact that these plants had no visible damage and suggesting that a mild loss of mitochondrial membrane potential may not result in obvious ROS accumulation. In contrast, plants treated with 15-min heat shock displayed strong mitoSOX fluorescence (Fig. 1D), suggesting that a burst of ROS had occurred in the mitochondria. This result is consistent with the severe loss of mitochondrial membrane potential in these plants and the lack of their recovery from the heat shock, indicating that this ROS burst may have caused severe damages to mitochondria. Plants treated with 30-min heat displayed a high background of auto-fluorescence, although mitoSOX fluorescence was not as strong as in plants treated with 15-min heat (Fig. 1D). We reasoned that since these plants were withered after the 30-min harsh treatment, cells were mostly dead and dehydrated at the time of staining and therefore their mitochondria could not be stained. Alternatively, mitochondrial ETC in these dying/dead cells might have ceased to function and therefore could not continue to generate ROS.

Mitochondria have a distinctive inner membrane cristae structure (Cogliati et al. 2016). The observation that acute heat shock significantly reduced mitochondrial membrane potential and increased ROS generation suggested that the stress may have led to oxidization of mitochondrial protein and lipid components and impaired membrane structure. To this end, we used transmission electron microscope (TEM) to evaluate the detrimental effect of the acute heat shock on mitochondrial ultrastructure. After analyzing > 200 mitochondria in plants treated with 5-min heat, we did not find any obviously defective cristae structure when compared with those from untreated plants (Fig. 1E). In contrast, a large portion of the mitochondria (83 out of 121 mitochondria examined) in plants treated with 15-min heat contained disrupted mitochondrial ultrastructure, such as loss of cristae and low electron density as indicated by the lighter stained matrix (Fig. 1E). In plants treated with 30-min heat shock, cellular membrane structures, including those of mitochondria, were severely disintegrated (Fig. 1E).

To further analyze the role of CL in plant cell response to stress, we checked subcellular CL distribution in heat-treated plants stably expressing the mitochondrial matrix-localized marker COX4-YFP, using the common fluorescent dye for CL, 10-N-nonyl-acridine orange (NAO) (Kaewsuya et al. 2007; Kholina et al. 2023). Two NAO staining patterns were observed: some overlapped with the mitochondrial matrix protein COX4-YFP (Fig. 1F), while others formed a ring around COX4-YFP (Fig. 1G). The ring-like distribution pattern resembles that of the mitochondrial outer membrane proteins like UBP27 (Pan et al. 2014b), suggesting that CL may have externalized to the outer membrane. Interestingly, the ring-like distribution of CL was often accompanied by vesicle-like structures with strong NAO staining but weak or no COX4-YFP signals, suggesting that these vesicles were enriched in CL, but contained little or no mitochondrial matrix proteins (Fig. 1G). It is likely that these vesicles are involved in CL relocation to other subcellular membranes away from the mitochondria.

In untreated plants, 93.3% (488 out of 523) of the cells analyzed showed mitochondrion-overlapping CL distribution (Fig. 1H). Heat treatment increased the portion of cells containing ring-like CL distribution to 22.2% (96 out 432 cells) after 5-min heat, and 93.5% (290 out of 310 cells) after 15-min heat, indicating that acute heat shock induces CL redistribution in *Arabidopsis* mitochondria (Fig. 1H). Plant cells with 30-min treatment showed strong auto-fluorescence and not NAO staining, possibly as an indication of cell death.

After recovery, plants treated with 5-min heat mostly resumed the mitochondrion-overlapping CL distribution pattern, i.e., 60.7% (218 out of 359 cells) after 1 h, 97.0% (292 out of 301 cells,) after 2 h, and 86.4% (153 out of 177 cells) after 24 h of recovery (Fig. 1H). However, plant cells with 15-min heat treatment still mostly remained the ring-like CL distribution pattern: 85.8% (313 out of 365 cells) after 0.5 h, 95.7% (200 out of 209 cells) after 1 h, and 74.9% (143 out of 191 cells) after 12 h of recovery (Fig. 1H). The plant cells could not be stained by NAO after 24 h of recovery, as the plants were mostly withered. Hence, whether or not heat shock-induced CL redistribution in plant cells can be reversed depends on the severeness of the heat damage.

In animal cells, CL redistribution is triggered by oxidative stress resulting from mitochondrial dysfunction (Chu et al. 2013; Garcia Fernandez et al. 2002). To estimate the integrity of mitochondrial function in cells showing different CL distribution patterns, we simultaneously stained the same leaf with NAO and TMRM, and observed that mitochondria with ring-like CL distribution had weaker TMRM, but stronger NAO signals (Fig. 1I). The lower MIMP was likely caused by impaired respiratory ETC, and the stronger NAO signal was possibly resulted from the externalized CL that was more easily stained, further supporting the conclusion that CL redistribution is correlated with mitochondrial dysfunction.

The acute heat shock caused serious alterations of mitochondria, including reduced MIMP, accumulation of mitochondrial ROS, disruption of mitochondrial ultrastructure, and CL redistribution. The level of mitochondrial alterations is correlated with the extent of the imposed heat shock. When the heat-induced mitochondrial alterations reach a certain level, e.g., a strong burst of mitochondrial ROS, extensive CL externalization, strong loss of MIMP and significant disruption of mitochondrial ultrastructure, the plants cannot recover. The extensive CL redistribution after heat treatment very likely represents a late stage of mitochondrial stress that will ultimately lead to cell death.

We discovered a novel type of mitochondrial derived vesicle-like structure that is CL enriched and most likely stress induced. These structures may be involved in stress response by removing CL from mitochondria. Our findings of stress-induced CL redistribution are generally in agreement with those revealed in animal cells, in which external stimuli hamper mitochondrial respiration and result in mitochondrial oxidative stress, causing CL peroxidation and redistribution to the organelle surface (Chu et al. 2013; Garcia Fernandez et al. 2002; Wiswedel et al. 2010). It is possible that CL externalization in plant mitochondria also function as a signal to activate downstream stress response pathways, such as mitophagy and PCD (Pizzuto and Pelegrin 2020; Acoba et al. 2020; Dudek 2017). The signaling pathways of mitophagy and PCD are not well characterized in plants. The protein regulators of plant mitophagy are recently emerging (Duckney et al. 2024), and ER is likely involved (Li et al. 2022). Heat stress was recently found to induce quick and extensive changes in mitochondrial and cytosolic protein abundance, with the release of mitochondrial proteins into the cytosol upon PCD induction (Schwarze et al. 2023). The exact role of CL in these pathways needs to be investigated in the future. It is also unclear whether other known vesicle-related mechanisms, such as the endomembrane trafficking or retromer complex (Li et al. 2023; Hu et al. 2022; Shen et al. 2013), are involved in CL related mitochondrial vesicle structure formation.

## Materials and methods

### Plant material and heat treatment

The normal growth condition for *Arabidopsis thaliana* in this study is 22 °C, 70% humidity, and 70–80 µmol m^−2^ s^−1^ white light for 14 h per day. Col-0 plants were used as the wild type. The mitochondrial marker line COX4–YFP was obtained from the Arabidopsis Biological Resource Center (ABRC). Plants were grown on petri dishes containing half-strength Linsmaier and Skoog medium. For heat treatment, the petri dishes containing plants were kept at 65 °C for various lengths of time, followed by recovery at normal growth conditions.

### Fluorescent chemical staining

NAO stock solution (2 mg/mL) was prepared in 100% ethanol, and the NAO working solution (5 µg/mL) prepared in deionized water was used to incubate 2-week-old seedlings for 10 min at room temperature. To minimize stress induced by long-time microscopy operation, CL distribution was observed under the microscope within a very short period of time (e.g. < 15 min).

TMRM staining was performed for observation of mitochondrial membrane potential. A stock solution (0.1 mg/mL) was prepared in DMSO, and working solution (1 µg/mL) prepared in deionized water was used to incubate seedlings for 10 min at room temperature.

For observation of mitochondrial ROS, MitoSOX™ Red Mitochondrial Superoxide Indicator staining was performed as previously described (Deng et al. 2014). A working solution of MitoSOX (5 μM) was used to incubate 2-week-old seedlings for 10 min at room temperature.

## Data Availability

All data generated or analyzed during this study are available from the corresponding author upon reasonable request.
